# The utility of metabolomics as a tool to inform maize biology

**DOI:** 10.1016/j.xplc.2021.100187

**Published:** 2021-04-21

**Authors:** David B. Medeiros, Yariv Brotman, Alisdair R. Fernie

**Affiliations:** 1 Max Planck Institute of Molecular Plant Physiology, Potsdam, Germany; 2 Department of Life Sciences, Ben-Gurion University of the Negev, Beersheva, Israel

**Keywords:** GWAS, metabolism, metabolite profiling, primary metabolites, secondary metabolites, *Zea mays*

## Abstract

With the rise of high-throughput omics tools and the importance of maize and its products as food and bioethanol, maize metabolism has been extensively explored. Modern maize is still rich in genetic and phenotypic variation, yielding a wide range of structurally and functionally diverse metabolites. The maize metabolome is also incredibly dynamic in terms of topology and subcellular compartmentalization. In this review, we examine a broad range of studies that cover recent developments in maize metabolism. Particular attention is given to current methodologies and to the use of metabolomics as a tool to define biosynthetic pathways and address biological questions. We also touch upon the use of metabolomics to understand maize natural variation and evolution, with a special focus on research that has used metabolite-based genome-wide association studies (mGWASs).

## Teaser

The maize metabolome is incredibly dynamic in terms of topology and subcellular compartmentalization. This review overviews a broad range of studies that cover recent developments in maize metabolomics, focusing on current methodologies and the use of metabolomics to define metabolic pathways and address relevant biological questions.

## Introduction

Maize (*Zea mays* L.), was first domesticated about 9,000 years ago from its wild relative, the lowland grass teosinte, in southwestern Mexico (Matsuoka et al., 2002; Piperno et al., 2009). In recent decades, it has become the most widely cultivated grain in the world, with a global production of about one billion metric tons in 2018 (Food and Agriculture Organization Corporate Statistical Database, http://www.fao.org/faostat/en/). Despite a loss of genetic diversity upon domestication, modern maize remains relatively rich in genetic variation, facilitating its cultivation in diverse environmental conditions (Vigouroux et al., 2002). The popularity of maize results not only from an increase in direct human consumption as food but also from the ever-increasing production of corn bioethanol, farm animal feed, and additional products such as syrup, oil, and cornmeal. Maize research, driven to a great extent by the crop's growing economic importance, has also risen dramatically. Several aspects of maize biochemistry, genetics, physiology, and ecology have been thoroughly explored in recent years, but needless to say, many knowledge gaps remain.

The rise of high-throughput genomic, transcriptomic, proteomic, and metabolomic tools constitutes one of the hallmarks of modern biological research. Maize has profited from the emergence of omics tools, with numerous recent studies delving into its systems biology. Given that maize and its products are used as both food and bioethanol, its metabolism has been given special attention. Several aspects of maize metabolism have received considerable attention, including (1) the role of the metabolome in the context of its participation in basic molecular processes and in responses to biotic and abiotic stresses and beneficial biotic interactions; (2) the nutritional composition of maize kernels and the molecular mechanisms that underlie the production of specific metabolites; (3) the means by which the metabolome and metabolic models link to leaf physiology and crop yield; (4) the metabolic alterations brought about by genetic modifications; and (5) the extent of natural variation in metabolism and its potential utility in breeding efforts. In addition, several further questions that cannot be strictly categorized have been addressed by maize metabolomics in recent studies on how pesticides influence the maize metabolome (Blondel et al., 2016); how phloem sap metabolites correlate with kernel yield (Yesbergenova-Cuny et al., 2016); how exposure of maize to polycyclic aromatic hydrocarbons (toxic organic pollutants) affects the metabolome (Sivaram et al., 2019); and the metabolic mechanisms that underlie plant root growth stimulation by smoke (Çatav et al., 2018).

Taking this broad basis into account, in this review we discuss both recent advances and trends in maize metabolomics, focusing on methodologies and on the contribution of metabolomics to defining metabolic pathways and addressing relevant biological questions. Finally, we discuss the identification of key genes that control maize metabolism.

## Methodologies

The maize metabolome is analyzed using essentially the same variety of methods used in other plant metabolomics studies, namely hyphenated mass spectrometry (MS) and nuclear magnetic resonance (NMR) (Obata and Fernie, 2012). Aside from several techniques rarely used in maize, such as infrared spectroscopy (Pavlík et al., 2010), two major approaches dominate: MS (e.g., Václavík et al., 2013) and NMR (e.g., Çatav et al., 2018). The former is more sensitive, whereas the latter can better quantify metabolites and detect conformational isomers (Obata and Fernie, 2012; Alseekh and Fernie, 2018). Although only a few research groups (Barros et al., 2010; Walker et al., 2011; Marti et al., 2013; Vinci et al., 2018) are able to use both approaches in parallel, it is generally agreed that they complement and enhance one another, with several studies dedicated to demonstrating just that (Venkatesh et al., 2016). MS tends to be the method of choice in most maize studies. MS/MS (tandem MS), an extended form of MS that uses ion fragmentation to enable superior identification, is increasingly used in maize metabolomics (Mesnage et al., 2016; Cocuron et al., 2019). Blondel et al. (2016) represents an example of the application of a little-used NMR variation, high-resolution magic-angle spin (^1^H-HRMAS), that enables metabolite detection in heterogeneous tissues or solutions without any extraction. In this case, the toxic effects of organochlorine pesticides in maize root tips were assessed by ^1^H-HRMAS, revealing profound alterations in the glycolysis/gluconeogenesis balance, inactivation of the tricarboxylic acid (TCA) cycle, and changes in internal nitrogen distribution, indicating that ^1^H-HRMAS NMR metabolomics can be a sensitive tool for understanding molecular disturbances.

In the most common metabolomics approaches used in maize, namely hyphenated MS methods, samples are separated into their components by gas chromatography (GC), liquid chromatography (LC), and frequently both, either in the same study, e.g., Tang et al. (2017), or in consecutive studies, e.g., Asiago et al. (2012) and its follow-up (Baniasadi et al., 2014); by capillary electrophoresis (CE) (Levandi et al., 2008; Leon et al., 2009); or, less ideally, by direct injection without prior separation (García-Flores et al., 2012). Although chromatography requires relatively lengthy extraction (and, in the case of GC, derivatization to render the metabolites volatile) in return for highly detailed results, direct injection provides rough profiles that are mainly useful for comparative purposes; however, the approach does have the advantage of requiring minimal preparation.

GC-MS is widely used for plant metabolomics and facilitates the identification and robust quantification of a few hundred metabolites in plant samples. Among these, sugars, sugar alcohols, amino acids, organic acids, and polyamines are often annotated, resulting in relatively comprehensive coverage of central primary metabolism. The great advantage of this approach is that highly stable protocols have been established for the setup and maintenance of machines and the evaluation and interpretation of chromatograms, meaning that libraries of retention times and mass spectral data for standard compounds can be shared among laboratories (Schauer et al., 2005). However, the use of GC-MS is limited to thermally stable volatile, or at least volatilizable, compounds. By contrast, LC does not require prior sample treatment and separates the components in a liquid phase. LC can analyze a variety of metabolites based on their chemical properties and the choice of columns, which include reversed phase, ion exchange, and hydrophobic interaction columns (Obata and Fernie, 2012). A crucial advantage of LC-MS is that, taking advantage of a variety of methods, we can analyze a wide array of metabolites, including those with high molecular mass and low thermostability. On the other hand, this flexibility presents difficulties in establishing mass spectral libraries for peak identification because mass spectra and retention times are dependent on instrument type (Moco et al., 2006). Finally, CE can separate a diverse range of chemical compounds and is more powerful than LC with respect to separation efficiency. One of the unique properties of CE-MS is the small amount of sample required for analysis; only nanoliters of sample are introduced into the capillary. A downside of CE is the poor migration time reproducibility and the absence of reference libraries, which can be only partially overcome by the prediction of migration time (Zhang and Ramautar, 2020).

Aside from these canonical methods generally used for non-targeted profiling, specific classes of metabolites lend themselves to analysis by other methods. For instance, carotenoids have been analyzed with a high-performance liquid chromatography (HPLC) photodiode array detector (Owens et al., 2014), used in this case for an association mapping study that correlated carotenoid levels with kernel color. In Decourcelle et al. (2015), carotenoids were profiled using HPLC, while at the same time the general metabolome was profiled using GC-MS. Similarly, targeted provitamin A and tocopherol determination protocols based on ultraperformance liquid chromatography (UPLC) have been used to screen maize germplasm (Wang et al., 2018a; Zhan et al., 2019).

The spatial distribution of metabolites within organisms has been an intriguing topic for decades, with implications related to biochemistry, kinetics, flux analysis, and physiology in general. In the past decade, however, matrix-assisted laser desorption-ionization MS imaging (MALDI-MSI) has been used as an analytical tool to visualize metabolites directly on plant tissues, and it has undergone important technical improvements in resolution, sensitivity, and chemical versatility (Sturtevant et al., 2016). This technique has been used to image maize metabolites at the cellular and subcellular levels in leaves (Korte et al., 2015; Dueñas et al., 2016, 2017), seeds (Feenstra et al., 2017a), and roots (Feenstra et al., 2017b; O'Neill and Lee, 2020). The same group also reported that they were able to achieve non-targeted profiling using MALDI-MSI, overcoming one of the main limitations of the method (Feenstra et al., 2015).

## Defining biochemical pathways

### Primary metabolism

Metabolites are traditionally divided into primary metabolites, which promote cell viability, and secondary/specialized metabolites, which contribute to the organism's viability in its environment. Primary metabolism not only plays a direct and pivotal role in plant growth, development, and reproduction but also produces precursors for secondary metabolite biosynthesis. Although some studies focusing on primary metabolites have used metabolomics as an important supportive tool, others have placed it at their core. Examples in which metabolomics has been used as a supportive analysis to reveal metabolic alterations in primary metabolism include the NMR-based profiling of two glutamine synthetase mutants, which showed rearrangement of nitrogen pathways that may affect lignin biosynthesis and hence underlie ear development, kernel set, and kernel filling (Broyart et al., 2010); GC-MS-based metabolite profiling of endosperm from 6-phosphogluconate dehydrogenase mutants, which suggested shifts in redox-related metabolites and increases in sugars (Spielbauer et al., 2013); the metabolic characterization, via LC-MS/MS, of mutants that lack the oxalyl-CoA decarboxylase1 gene and undergo major metabolic changes in the endosperm (Yang et al., 2018a); GC-MS-based metabolite analysis of mutants of the important C_4_ enzyme pyruvate orthophosphate dikinase (PPDK), which showed that sugar signaling and nitrogen metabolism changed dramatically (Zhang et al., 2018); the metabolic characterization of the *closed stomata1* maize mutant, which defined *CST1* as a link in the feedback regulation of stomatal movement and photosynthesis (Wang et al., 2019a); and the establishment of genetic resources for the study of vitamin B-related metabolism in maize (Suzuki et al., 2020).

Metabolomics approaches have also been used as the main tool for deciphering the importance of the main pathways of primary metabolism. For instance, the responses of the leaf metabolome in different growth zones of maize leaves (cell division, elongation, and mature) and under carbon depletion were also investigated and linked to the rate of leaf elongation and protein synthesis. Central metabolism was shown to differ markedly between the growth and mature zones, and metabolic response during carbon depletion was less pronounced and was delayed in the growth zones compared with mature tissue. Interestingly, leaf growth largely followed sucrose content in the growth zones (Czedik-Eysenberg et al., 2016).

GC-MS-based metabolite profiling has been used to uncover the genetic basis for differences in primary metabolism and its relationship to plant performance in maize inbred populations (Wen et al., 2015, 2018; Cañas et al., 2017). In addition, a time-series metabolome analysis integrated with proteome data from maize hybrids and their inbred parents revealed that hybrids can better tolerate photoinhibition stress, maintaining higher photosynthesis without excessive elevation of photorespiration compared with the inbred lines. This highlights the roles of photosynthetic and photorespiratory pathways in maize seedling heterosis and provides advances for the biotechnological improvement of hybrid crops (Li et al., 2020b).

Maize uses a specialized photosynthetic pathway, C_4_ metabolism (Hatch, 1987), as opposed to most other plants, including grasses such as wheat and rice, which use C_3_ metabolism. C_4_ plants spatially divide their photosynthetic process into two cell types, the mesophyll and bundle sheath cells, thereby fixing CO_2_ and using water more efficiently (Schlüter and Weber, 2020). This allows higher yields in warmer climates; therefore, the integration of C_4_ traits into C_3_ plants to increase yield and environmental tolerance has been the new challenge. In fact, the installation of a C_4_ photosynthetic pathway in rice has been predicted to increase rice yields by up to 50% (Hibberd et al., 2008), and attempts to install a partial C_4_ pathway into rice using maize enzymes have been successfully metabolically confirmed using LC-MS methods (Ermakova et al., 2020; Lin et al., 2020). Comparative metabolic analyses combined with transcriptomic data from C_3_ (rice) and C_4_ (maize) plants were used either to identify differences between the two photosynthetic mechanisms (Wang et al., 2014a) or to draw the evolutionary histories of both groups (Deng et al., 2020). Arrivault et al. (2019) profiled the abundance of Calvin-Benson cycle (CBC) metabolites from five C_3_ plants (including rice) and four C_4_ plants (including maize). They discovered substantial differences not only between C_3_ and C_4_ groups but also within each group, especially among the five C_3_ species, suggesting independent evolution of CBC regulation in different plant lineages.

**Figure 1:**
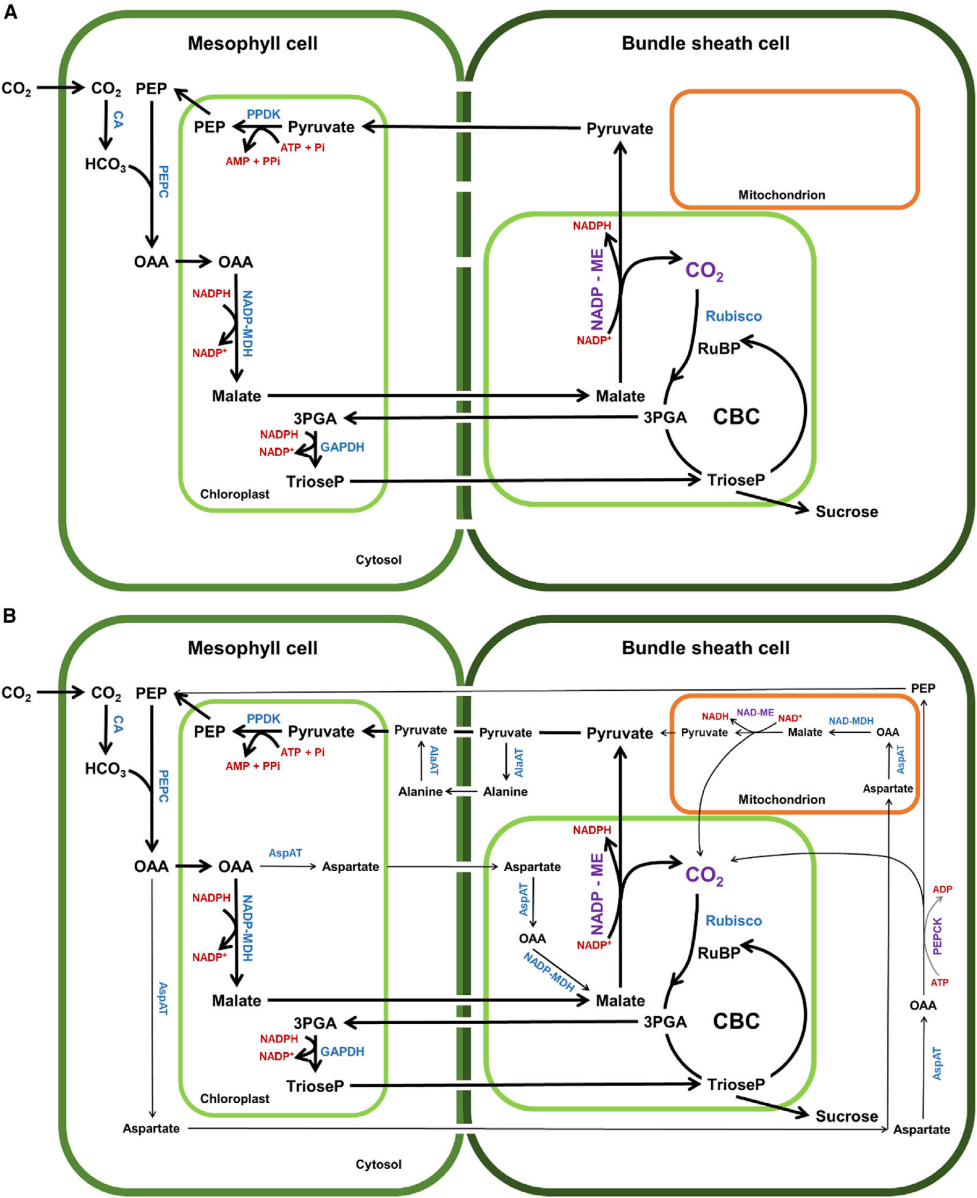
C_4_ metabolism model in maize **(A)** NADP-ME decarboxylation pathway. The first step in the C4 cycle is the assimilation of CO_2_ into oxaloacetate (OAA) by phospho*enol*pyruvate carboxylase (PEPC). In the NADP-ME cycle, OAA is imported into the chloroplasts of mesophyll cells (MCs) and reduced to malate. Malate diffuses along its concentration gradient into the bundle sheath cells (BSCs), where it is imported into the chloroplasts and decarboxylated by NADP-ME. This reaction yields one molecule each of CO_2_, reduced NADP (NADPH), and pyruvate. CO_2_ is assimilated by ribulose-1,5-bisphosphate carboxylase-oxygenase (Rubisco), yielding two molecules of 3-phosphoglyceric acid (3PGA) that can enter the CBC in either the BSCs or the MCs. The latter requires the shuttling of 3PGA and triose phosphate (TrioseP) between the BSCs and MCs. Pyruvate is exported from the BSCs to MCs and taken up into the chloroplasts, where it is converted to phosphoenolpyruvate (PEP) by PPDK. This reaction consumes adenosine triphosphate (ATP) and phosphate (Pi) and releases one molecule of adenosine monophosphate (AMP) and pyrophosphate (PPi). PEP is exported from the chloroplast and can enter a new cycle of the CO_2_ shuttle. **(B)** NADP-ME as the main decarboxylation pathway in combination with the NAD-ME and PEPCK pathways. The NAD-ME pathway involves the conversion of OAA into aspartate by aspartate aminotransferase (AspAT) in the MCs; it enters the BSCs and moves from there into the mitochondria. Aspartate is converted to OAA by AspAT and then reduced to malate by NAD-dependent malate dehydrogenase (NAD-MDH). Malate is oxidatively decarboxylated by mitochondrial NAD-ME, releasing reduced nicotinamide adenine dinucleotide (NADH), pyruvate, and CO_2_. CO_2_ enters the CBC as in the NADP-ME pathway, and pyruvate is converted into alanine in the cytosol by alanine aminotransferase (AlaAT). Alanine is exported to the MCs and deaminated to pyruvate by cytosolic AlaAT, and pyruvate is then used to regenerate the initial acceptor PEP, as in the NADP-ME cycle. The PEPCK cycle exhibits components of both the NADP-ME and NAD-ME cycles, but it has additional steps for decarboxylation and regeneration of the acceptor. In this cycle, part of the OAA from the initial CO_2_ assimilation by PEPC in the MCs is converted to aspartate, which is transferred to the BSCs. Aspartate is deaminated to OAA via cytosolic AspAT and subsequently decarboxylated by PEPCK in a reaction that consumes ATP. The products of this reaction are PEP and CO_2_. CO_2_ is assimilated by Rubisco as in the NADP-ME cycle, and PEP returns to the MCs as an initial acceptor. Abbreviations: ADP, adenosine diphosphate; CA, carbonic anhydrase; GAPDH, glyceraldehyde phosphate dehydrogenase; HCO_3_, bicarbonate; NADP-MDH, NADP-dependent malate dehydrogenase; RuBP, ribulose 1,5-bisphosphate.

C_4_ plants are traditionally classified into three distinct subtypes based on the enzyme that performs the primary decarboxylation reaction in the bundle sheath cells: plastidial nicotinamide adenine dinucleotide phosphate (NADP)-dependent malic enzyme (NADP-ME), mitochondrial nicotinamide adenine dinucleotide (NAD)-dependent malic enzyme (NAD-ME), and cytosolic phosphoenolpyruvate carboxykinase (PEPCK). Maize is predominantly categorized as the NADP-ME subtype (Figure 1A), but recent evidence indicates that the C_4_ cycle functions as a branched rather than a linear pathway (Figure 1B), providing flexibility between the different decarboxylation pathways that may be controlled by developmental and environmental cues (Pick et al., 2011; Wang et al., 2014b). Pick et al. (2011) showed that there is no evidence to suggest switches between the decarboxylation pathways within the age gradient of a single leaf, but a previous study (Wingler et al., 1999) reported higher activity of PEPCK in older leaves, pointing to the developmental regulation of decarboxylation pathways. This idea is supported by the metabolic characterization of maize DCT2 mutants, which show impaired malate transport into bundle sheath cells (Weissmann et al., 2016), and by following the carbon flux through C_4_ photosynthesis of maize (Arrivault et al., 2016). Both studies used isotopic labeling experiments combined with metabolomics approaches and concluded that the maintenance of different C_4_ decarboxylation pathways may robustly afford high photosynthetic efficiency under a broad range of environmental conditions.

### Secondary metabolism

Secondary metabolites are fascinating because of their chemical and functional diversity. For each way that the plant interacts with its environment, there are thousands of metabolites that serve as mediators. Thanks to clear chemical classification, many studies focus on a single class of metabolites, with specific goals. For a comprehensive review of carotenoids and anthocyanins in maize kernels, see Ranilla (2020). For instance, studies have focused on carotenoids, with the aim of increasing their abundance to help prevent dietary vitamin A deficiency (Owens et al., 2014); tocochromanols, with an identical aim concerning vitamin E (Lipka et al., 2013); and flavonoid derivates, for both their antioxidant power (Lago et al., 2014; Li et al., 2021) and their contribution to lignin deposition to improve biomass processing (Eloy et al., 2017).

Questions that accompany these main topics have also been studied. For instance, although maize that produces enhanced amounts of carotenoids was generated some time ago, the global effects of this alteration were only explored years later in a representative example of a triple-omics approach (Decourcelle et al., 2015). In the same vein, maize engineered for the production of astaxanthin, a rare and exceptionally desired carotenoid that does not occur naturally in maize, was metabolically profiled (Farré et al., 2016); certain perturbations in central metabolism were observed, and these were within the natural variation of the parental plants.

Biotic stress is a major constraint to productivity in modern maize varieties (also see section, “metabolomics of stress response”). In recent years, several studies have used quantitative genetics and metabolomics approaches to help identify several genes and secondary metabolic pathways involved in maize native resistance mechanisms to biotic threats (Table 1). For instance, maysin, the major C-glycosyl flavone in silks of most maize varieties, is synthesized in a branch of the flavonoid pathway and confers maize resistance to the corn earworm, an important insect pest in maize and other crops (Waiss et al., 1979; Elliger et al., 1980). Since its discovery, the genetic background for maysin biosynthesis has been extensively studied and was recently completely described (Casas et al., 2016). Interestingly, maize varieties adapted to high altitudes exhibited higher accumulation of maysin after UV-B exposure not only in silks but also in leaves (Casati and Walbot, 2005), suggesting that this specific environmental condition may trigger specific plant metabolic responses to biotic attacks (see section, “how combined stresses affect the maize metabolome”).

**Table 1:** Genetic mapping studies on metabolic traits in maize.

Trait	Measurement	Candidate gene, locus, or encoding enzyme	Analysis	Reference
Carotenoids in kernels	LC	*y1*, *vp5*, and QTL	linkage mapping	Wong et al. (2004)
Maysin and chlorogenic acid in silks	LC	*p*, *a1*, *c2*, and *whp1*	linkage mapping	Szalma et al. (2005)
Oleic acid in kernels	GC	*fad2*	linkage mapping	Beló et al. (2008)
Carotenoid composition and content in kernels	LC	*lcyE*	association and linkage mapping	Harjes et al. (2008)
Oil content and fatty acid composition in seeds and embryos	NMR and GC	*dgat1-2*	QTL mapping	Zheng et al. (2008)
β-carotene in grains	LC	*lcyE* and *crtRB1*	QTL and linkage mapping	Yan et al. (2010)
Oil content and fatty acid composition in kernels	GC	multiple candidate genes	QTL and linkage mapping	Yang et al. (2010)
Palmitic acid content in kernels	GC	*fatb*	QTL, association, and linkage mapping	Li et al. (2011)
Carbohydrates and ABA metabolites during stress in ears, silks, and leaves	ELISA and spectrophotometry	multiple candidate genes	association mapping	Setter et al. (2011)
Anthocyanin in kernels	LC	*f3′h1*	linkage mapping	Sharma et al. (2011)
Oil content and fatty acid composition in kernels	NMR and GC	*dgat1-2*	linkage mapping	Chai et al. (2012)
Starch, protein, and oil content in kernels	NIRS	multiple candidate genes	linkage mapping and GWAS	Cook et al. (2012)
α-tocopherol content in kernels	LC	*vte4*	linkage mapping and GWAS	Li et al. (2012)
Leaf metabolome	GC-MS	multiple candidate genes	GWAS	Riedelsheimer et al. (2012)
α-carotene in kernels	LC	*crtRB3*	QTL and linkage mapping	Zhou et al. (2012)
Carotenoid content in grains	LC	*psy1*	QTL and linkage mapping	Fu et al. (2013)
Carotenoid composition and concentration in grains	LC	multiple candidate genes	QTL and linkage mapping	Kandianis et al. (2013)
Oil biosynthesis in kernels	GC	multiple candidate genes	linkage mapping and GWAS	Li et al. (2013)
Tocochromanols in grains	LC	*hggt1* and GRMZM2G437912	GWAS	Lipka et al. (2013)
Aphid resistance/benzoxazinoid content in leaves	LC-MS	*bx10a*, *bx10b*, and *bx10c*	QTL and association mapping	Meihls et al. (2013)
Leaf lipidome	LC-MS	multiple candidate genes	GWAS	Riedelsheimer et al. (2013)
Carotenoids in kernels	LC	multiple candidate genes	GWAS	Owens et al. (2014)
Metabolic diversity of kernels	LC-MS	multiple candidate genes	linkage mapping and GWAS	Wen et al. (2014)
Carotenoids in kernels	LC	multiple candidate genes	GWAS	Suwarno et al. (2015)
Primary metabolism in leaves and kernels	GC-MS	multiple candidate genes	QTL and linkage mapping	Wen et al. (2015)
Carbon and nitrogen metabolism in leaves	spectrophotometry	multiple candidate genes	linkage mapping and GWAS	Zhang et al. (2015)
Ratio of tocotrienols^a^	LC	*vte1*	GWAS	Chen and Lipka, (2016)
Starch content in kernels	NIRS	multiple candidate genes	GWAS	Liu et al. (2016b)
Metabolic diversity in mature kernels	LC-MS	multiple candidate genes	QTL, linkage mapping, and GWAS	Wen et al. (2016b)
Carbohydrates and ABA metabolites during stress in ears, silks, and leaves^b^	ELISA and LS	multiple candidate genes	GWAS	Zhang et al. (2016)
Amino acids in kernels	CEC and spectrophotometry	multiple candidate genes	QTL, linkage mapping, and GWAS	Deng et al. (2017)
Root volatiles	GC-MS and GC-FID	*tps21*	QTL, linkage mapping, GWAS	Ding et al. (2017)
Flavonoid biosynthesis in kernels^c^	LC-MS	multiple candidate genes	linkage mapping and GWAS	Jin et al.( 2017)
Nitrogen metabolism in leaves	spectrophotometry	GRMZM2G008714 GRMZM2G045171 GRMZM2G082780 GRMZM2G088235 GRMZM2G180625	QTL mapping	Trucillo Silva et al. (2017)
Carotenoid content in kernels	LC	multiple candidate genes	GWAS	Azmach et al. (2018)
Tocochromanol content in kernels	LC	multiple candidate genes	QTL and linkage mapping	Fenton et al. (2018)
Nitrogen metabolism in roots	spectrophotometry	multiple candidate genes	QTL mapping	Trucillo Silva et al. (2018)
Tocopherol content in kernels	LC	multiple candidate genes	linkage mapping and GWAS	Wang et al. (2018a)
Primary metabolism in leaves and kernels	GC-MS	multiple candidate genes	GWAS	Wen et al. (2018)
Kernel composition and flour pasting behavior	NIRS	multiple candidate genes	GWAS	Alves et al. (2019)
Tocochromanols in kernels	LC	*vte1*, *vte4*, *hggt1*, *sh2*, *su1*	GWAS	Baseggio et al. (2019)
Diterpenoid defenses	GC-MS and LC-MS	multiple candidate genes	GWAS	Ding et al. (2019)
Oil and fatty acid composition in kernels^d^	GC	multiple candidate genes	GWAS and pathway analysis	Li et al. (2019a)
Primary metabolites in leaves and kernels	GC-MS and spectrophotometry	QTL	QTL and linkage mapping	Li et al. (2019b)
Starch content in kernels	NIRS	GRMZM2G110929 GRMZM5G852704	linkage mapping and GWAS	Lin et al. (2019)
Cell wall-bound hydroxycinnamates in stems	LC and spectrophotometry	multiple candidate genes	GWAS	López-Malvar et al. (2019)
Mechanisms of phosphorus deficiency in leaves and roots	LC-MS and GC-MS	GRMZM2G051806 GRMZM2G025854 GRMZM2G039588 GRMZM2G050570 GRMZM5G841893	GWAS	Luo et al. (2019)
Tocopherol content in leaves and kernels	ELISA and LC	*porb2*	QTL and association mapping	Zhan et al. (2019)
Antibiotic biosynthesis (zealexin)	GC-MS and LC-MS	multiple candidate genes	GWAS	Ding et al. (2020)
Secondary metabolites in leaves	LC-MS	multiple candidate genes	GWAS	Zhou et al. (2019)
Volatile composition in wholemeal flour	GC-MS	multiple candidate genes	GWAS	Alves et al. (2020b)
Antioxidant content in kernels	LC and spectrophotometry	multiple candidate genes	GWAS	Alves et al. (2020a)
Carotenoids in kernels	LC	multiple candidate genes	GWAS	Baseggio et al. (2020)
Carotenoids in kernels	HPLC	multiple candidate genes	linkage mapping and GWAS	Diepenbrock et al. (2020)
Anthocyanin in kernels	HPLC and spectrophotometry	multiple candidate genes	GWAS	Chatham and Juvik, (2021)
Metabolite biomarkers for salt tolerance	LC-MS	*cts3*, *cyp709b2*, *ugt*, and multiple candidate genes	GWAS	Liang et al. (2021)

Abbreviations: a1, anthocyaninless1; bx10a, benzoxazinoneless10a; bx10b, benzoxazinoneless10b; bx10c, benzoxazinoneless10c; c2, colorless2; CEC, cation exchange chromatography; *crtRB1*, β-carotene hydroxylase 1; *crtRB3*, β-carotene hydroxylase 3; cts3, citrate synthase 3; cyp709b2, cytochrome P450; DGAT1-2, acyl-CoA:diacylglycerol acyltransferase; *f3′h1*, flavonoid 3′-hydroxylase; fad2, fatty acid desaturases-2; *fatb*, acyl-ACP thioesterase; GC, gas chromatography; GC-FID, gas chromatography-flame ionization detector; GRMZM2G008714, pyruvate kinase; GRMZM2G025854, phosphoglucomutase; GRMZM2G039588, glucose-6-phosphate 1-epimerase; GRMZM2G045171, sucrose synthase; GRMZM2G050570, threonine synthase; GRMZM2G051806, hexokinase; GRMZM2G082780, phosphoenolpyruvate carboxylase 4; GRMZM2G088235, urease protein; GRMZM2G110929, GLABRA2 expression modulator; GRMZM2G180625, glyceraldehyde-3-phosphate dehydrogenase; GRMZM2G437912, paralog gene encoding a prephenate dehydratase; GRMZM5G841893, FAD-dependent urate hydroxylase; GRMZM5G852704, ethylene-responsive transcription factor RAP2-4; hggt1, homogentisate geranylgeranyltransferase; LC, liquid chromatography; LC-MS, liquid chromatography-mass spectrometry; lcyE, lycopene epsilon cyclase; NIRS, near-infrared spectroscopy; p, pericarp color; porb2, protochlorophyllide oxidoreductase; *psy1*, phytoene synthase 1; QTL, quantitative trait loci; sh2, shrunken2; su1, sugary1; tsp21, terpene synthase21; ugt, glucosyltransferase; vp5, viviparous 5; *vte4*, γ-tocopherol methyltransferase; whp1, white pollen1; y1, yellow 1.

a Metabolic data taken from Owens et al. (2014) and Lipka et al. (2013).

b Metabolic data taken from Setter et al. (2011).

c Metabolic data taken from Wen et al. (2014) and Wen et al. (2016b).

d Metabolic data taken from Li et al. (2013).

Maize also produces a range of herbivore-induced terpene volatiles and pathogen-induced non-volatile terpenoids that play significant defensive and developmental roles. Terpenoids, also known as isoprenoids, originate from the conjugation of dimethylallyl diphosphate and its isomer isopentenyl diphosphate. Subsequent reactions catalyzed by prenyl transferases and prenyl diphosphatases yield the different classes of terpenes. Rearrangements of terpene molecules by terpene synthases (TPSs) and cytochrome P450 enzymes result in a myriad of terpenoids (Tholl, 2015). Recent studies have characterized about half of the 30 TPS genes present in the maize genome (reviewed by Block et al., 2019). The volatile products of TPSs have been detected in different maize tissues, but their composition and content depend strongly on the genetic background (Degen et al., 2004), developmental stage, and organ (Köllner et al., 2004), as well as on abiotic and biotic stress (Gouinguené and Turlings, 2002; Becker et al., 2014; Block et al., 2017; Chiriboga et al., 2018). Upon biotic attack, maize plants can also produce different classes of non-volatile terpenoid phytoalexins. For instance, kauralexins and dolabralexins are two major diterpenoid groups that have important roles in responses to biotic stress. Kauralexins mediate defense responses against fungal pathogens (Christie et al., 2017; Meyer et al., 2017; Christensen et al., 2018b) and insect herbivory (Dafoe et al., 2011), whereas dolabralexins not only inhibit pathogenic fungi (Mafu et al., 2018) but also affect the rhizosphere microbial community (Murphy et al., 2021). Zealexins, non-volatile sesquiterpenoid phytoalexins, are also elicited in response to herbivory (Christensen et al., 2018a) and infection with diverse fungal pathogens (Basse, 2005; Köllner et al., 2008; Huffaker et al., 2011; Christensen et al., 2018a). Interestingly, the accumulation of zealexin A4 seems to be attenuated by high CO_2_ levels, supporting the idea that elevated CO_2_ has a negative impact on maize chemical defense against biotic stress (Vaughan et al., 2014, 2016).

**Figure 2:**
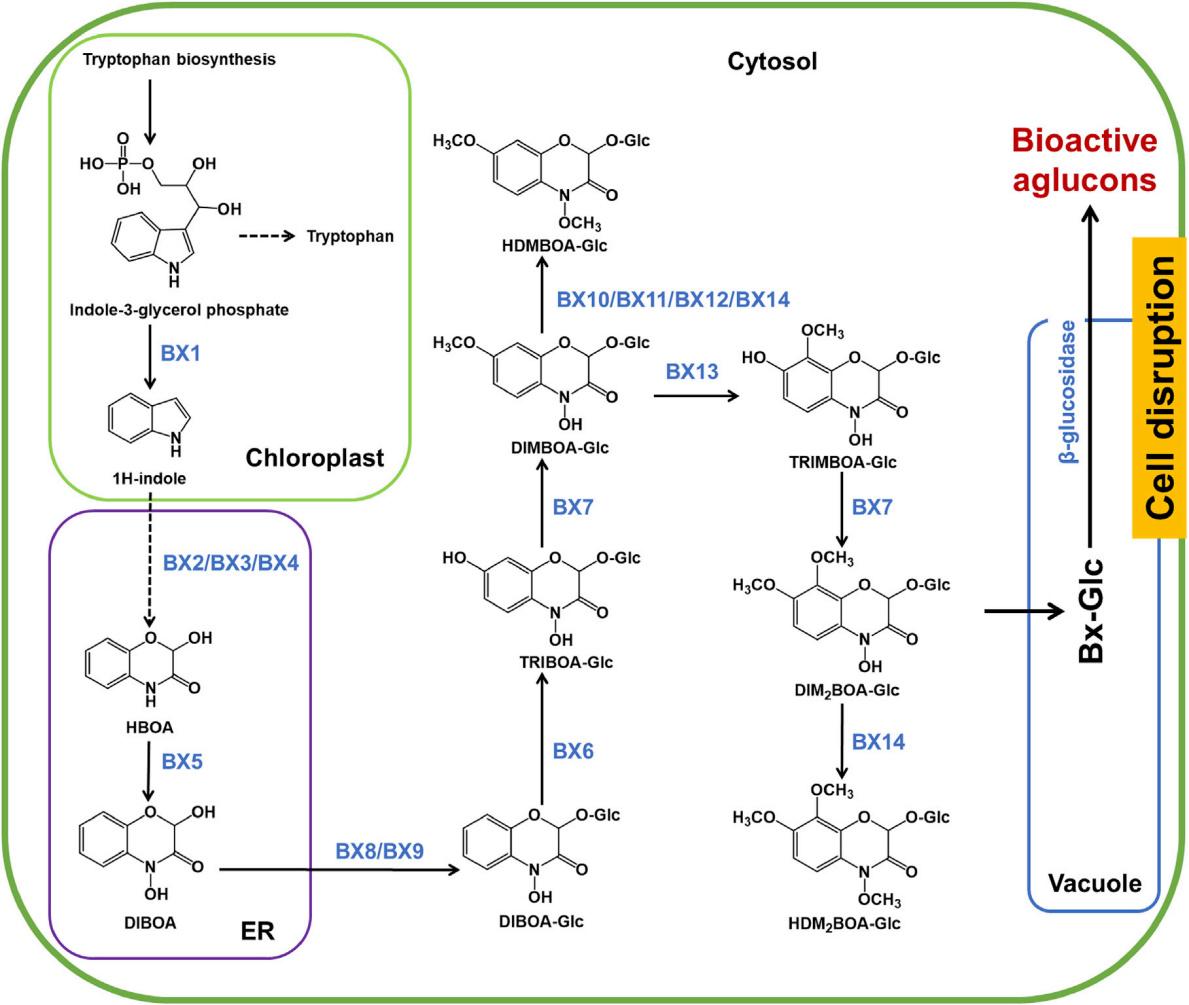
Benzoxazinoid biosynthetic pathway in maize Benzoxazinoid biosynthesis begins in the chloroplast by the conversion of indole-3-glycerol phosphate (an intermediate of tryptophan biosynthesis) into indole, catalyzed by the indole-3-glycerol phosphate lyase benzoxazinoneless 1 (BX1). A subsequent stepwise introduction of four oxygen atoms by the P450 monooxygenases BX2, BX3, BX4, and BX5 leads to the formation of 2,4-dihydroxy-1,4-benzoxazin-3-one (DIBOA). DIBOA is a substrate for the UDP-glucosyltransferases BX8 and BX9, which convert the toxic compound DIBOA into the stable glucoside (Glc) form DIBOA-Glc. The 2-oxoglutarate-dependent dioxygenase (2ODD) BX6 catalyzes a hydroxylation of DIBOA-Glc at C-7, followed by a methylation of the introduced hydroxyl group catalyzed by the *O*-methyltransferase BX7, yielding DIMBOA-Glc in the cytosol. An *O*-methylation reaction catalyzed by a group of three *O*-methyltransferases (BX10, BX11, and BX12) converts DIMBOA-Glc to 2-hydroxy-4,7-dimethoxy-1,4-benzoxazin-3-one glucoside (HDMBOA-Glc). BX13, a BX6-like 2-ODD, catalyzes the conversion of DIMBOA-Glc to 2,4,7-trihydroxy-8-methoxy-1,4-benzoxazin-3-one glucoside (TRIMBOA-Glc). TRIMBOA-Glc can be *O*-methylated by BX7 to form 2,4-dihydroxy-7,8-dimethoxy-1,4-benzoxazin-3-one glucoside (DIM_2_BOA-Glc), which can be further methylated by the *O*-methyltransferase BX14 to generate 2-hydroxy-4,7,8-trimethoxy-1,4-benzoxazin-3-one glucoside (HDM_2_BOA-Glc). BX14 can also produce HDMBOA-Glc from DIMBOA-Glc. The benzoxazinoid glucosides (Bx-Glc) are stored in the vacuole, where they are protected from β-glucosidases located in the chloroplast and cell wall. Upon cell disruption (e.g., by herbivory), the Bx-Glc are exposed to the β-glucosidases, which cleave the glucosyl group, generating bioactive aglucons. Abbreviations: ER, endoplasmic reticulum; TRIBOA-Glc, 2,4,7-trihydroxy-1,4-benzoxazin-3-one glucoside.

Grasses synthesize a unique class of secondary metabolites known as benzoxazinoids. They are among the most agriculturally relevant groups of plant specialized metabolites because, since their identification in the 1950s, benzoxazinoids have been associated with defense against insect herbivores, microbial pathogens, and competing plant species. Recently, they have also been associated with signaling events (Zhou et al., 2018). These ∼20 natural defense chemicals share the 2-hydroxy-2*H*-1,4-benzoxazin-3(4*H*)-one skeleton (HBOA) and are found mainly as inactive glucoside-bound precursors stored in the vacuole (Frey et al., 2009). The core biosynthetic pathway of the major maize benzoxazinoid (Figure 2), 2-(2,4-dihydroxy-7-methoxy-1,4-benzoxazin-3-one)-β-d-glucopyranose (DIMBOA-Glc), has been characterized (reviewed by Zhou et al., 2018). However, only recently have later biosynthetic steps been characterized in maize (Handrick et al., 2016), and the transcriptional regulation of the biosynthetic pathway has only begun to be revealed (Zhang et al., 2021). Upon herbivore-mediated tissue damage, glucosidases cleave the glucoside moiety of the benzoxazinoid glucosides, producing biocidal aglucone benzoxazinoids (Morant et al., 2008). It has also been proposed that benzoxazinoids such as 6-methoxy-benzoxazolin-2-one (MBOA) and 2,4-dihydroxy-7-methoxy-1,4-benzoxazin-3-one (DIMBOA) may regulate belowground and aboveground biotic interactions (Meihls et al., 2013; Hu et al., 2018). In fact, benzoxazinoids were shown to affect microbial communities in shoots and roots, as well as the rhizosphere (Kudjordjie et al., 2019). The negative correlation between benzoxazinoids and fungal pathogen genera in shoots could indicate a potential for these compounds to serve as a control for pathogenic fungal infections. Further evaluation of the regulatory activity of benzoxazinoids on the maize metabolome and associated microbial communities revealed that benzoxazinoids influence the rhizobiome through the endogenous regulatory activity of plant-derived rhizosphere signals such as flavonoids (Cotton et al., 2019). Benzoxazinoids are often identified by targeted HPLC profiling, but in-depth studies that attempted to gain comprehensive insights into metabolic alterations upon herbivore attack have used non-targeted approaches (Glauser et al., 2011). In this respect, these studies and those that define the pathways of flavonoid biosynthesis (Wen et al., 2020) are distinct from studies of primary metabolism, as they define the pathway structure—and in some cases the function—of the metabolite rather than the state of metabolic regulation, and such information is still lacking for many secondary metabolites.

### Lipids and oils

Although starch is the most commercially and nutritionally interesting component of maize, its oil is gaining importance for cooking, as a component of foods such as margarine, and for products such as soap, ink, and paint. The oil content of maize is low, about 3%, but it can reach up to 7% in high-oil corn genotypes (Singh et al., 2014). Maize seeds possess high oleic acid contents and, although it is beneficial to human health, oleic acid is sensitive to oxidation and unstable at high temperatures (Du et al., 2016). Attempts have been made to increase oil content, with metabolomics playing an important confirmatory role (Pouvreau et al., 2011). Increased oil production in maize is also desirable for reasons other than direct use of the oil. Indeed, the astaxanthin-producing maize line was crossed with a high-oil-producing line to facilitate the storage and easy extraction of the lipophilic carotenoid (Farré et al., 2016). Moreover, the fatty acid composition of maize oil has also been a target for manipulation for a variety of nutritional and commercial purposes (White et al., 2007; Du et al., 2016), and, as discussed below, genetics continues to be an important tool for the identification of breeding targets.

## Using metabolomics to address biological questions

### Metabolomics of stress response

During their lives, plants can experience unfavorable growth conditions due to biotic and abiotic stresses that can retard their growth and impair productivity. In these unfriendly growth environments, plants need to adapt to survive. In such scenarios, plant metabolism is perturbed, and the metabolic network must be reprogrammed. Metabolomics is a powerful tool for gaining a comprehensive perspective on how metabolic networks are regulated, and it has been used extensively in maize stress response research. It is established that plants adjust their metabolite production in response to stress, but the reasons, mechanisms, and regulation are only partially known. Common stresses that plants experience in nature include herbivory and pathogen infection, as well as water-deficit stress, toxicity (including salinity), nutrient deficiency, radiation, low or high temperatures, and excess or deficient light. All these stresses have been studied under both field and controlled conditions, frequently showing that the responses in controlled growth conditions are not always faithful replicates of those in the field (Casati et al., 2011; Witt et al., 2012). Below we summarize the most recent findings on stress metabolomics studies that directly address the following questions.

#### Which organs are most affected?

Leaf blades were demonstrated to be the sites of greatest metabolic change following drought (Witt et al., 2012; Obata et al., 2015). Under high-salinity conditions, shoots were found to be more metabolically affected than roots, with effects similar to those caused by osmotic stress (Gavaghan et al., 2011). Likewise, comparative metabolite profiling of low-phosphorus (P)-tolerant and low-P-sensitive maize genotypes also revealed that leaves seem to be the main site for metabolic changes under phosphorus starvation (Ganie et al., 2015). Engineered nanomaterials, increasingly used in soil remediation, constitute an interesting potential niche stressor, but their impact on plant physiology remains obscure. A study in maize showed considerable metabolic alterations, and although roots were the site of exposure to these nanomaterials, metabolite changes were also pronounced in leaves (Zhao et al., 2019; Li et al., 2020a; Yan et al., 2020).

When leaves are infested by the African cotton leafworm moth, many metabolite changes are observed at the site of infection; defense-related metabolites increase in the vascular sap and root exudates, but only a few metabolites are changed in the roots (Marti et al., 2013). The effect of the smut caused by the fungus *Ustilago maydis* on maize root metabolism has been characterized in detail (Djamei et al., 2011). Among the 150 proteins with known functions in the *U. maydis* secretome, chorismate mutase seems to be a virulence factor. It has been suggested that chorismate mutase enters the plant cell and channels chorismate into the phenylpropanoid pathway, preventing its flow toward salicylic acid biosynthesis, probably as a mechanism to reduce maize resistance to *U. maydis*. This illustrates a reprogramming of maize root metabolism by a fungal effector to favor the fungus’s requirements. The suppression of salicylic acid by *U. maydis* was further demonstrated by the identification of a cytoplasmic *U.  maydis* salicylate hydroxylase that is induced during plant colonization (Rabe et al., 2013). Another *U. maydis* secreted effector, Tin2, was shown to induce changes in anthocyanin biosynthesis (Tanaka et al., 2014). Tin2 translocates into plant cells and targets the cytoplasmic protein kinase ZmTTK1, which is stabilized, leading to anthocyanin formation as a strategy to compete with lignification of the colonized tissue. This was later characterized as a neofunctionalization of Tin2, probably related to the rare ability of *U. maydis* to induce leaf tumors by lowering lignification that might otherwise restrict fungal proliferation (Tanaka et al., 2019).

#### Which pathways are mainly affected?

Alvarez et al. (2008) were among the pioneers in studying the maize stress response using metabolomics, particularly HPLC-MS/MS fragmentation. Because no libraries existed at the time, the authors screened their results against 64 standards of compounds thought to exist in the xylem sap. Changes were found in hormones associated with stomatal movements, such as abscisic acid (ABA) and cytokinins, highlighting the functioning of root-to-shoot signals. In addition, increases in the content of phenylpropanoid pathway intermediates were also observed. Although this is probably related to impacts on lignin and anthocyanin biosynthesis under water stress, it may also affect flavonol levels with effects on stomatal aperture, as suggested for other species (reviewed by Medeiros et al., 2020). In fact, just recently, Li et al. (2021) showed that increased flavonol content in guard cells improves the water use efficiency of a drought tolerant maize genotype (*doi57*; *drought overly insensitivity 57*) by both increasing antioxidant capacity and downregulating stomatal closure in the mutant plants under drought stress.

Schlüter et al. (2013) found similarities in the regulation of carbon metabolism in source leaves under low temperature and low nitrogen stress and corresponding impacts on plant growth. Accumulation of carbohydrates under these two conditions indicated that growth was limited by a feedback downregulation of photosynthesis. Moreover, phosphorus deficiency directly influenced carbon and energy metabolism: photosynthesis dropped dramatically, and a decrease in carbohydrate levels was observed. However, nitrate assimilation was the only primary pathway downregulated under all three conditions (low temperature, low nitrogen, and low phosphorus stress). The coordination of carbon and nitrogen metabolism is known to affect plant growth, and a mixed supply of nitrate (NO_3_^–^) and ammonium (NH_4_^+^) can maximize plant growth compared with a sole NO_3_^–^ or NH_4_^+^ supply. However, only recently, Wang et al. (2019b) shed light on this observation by showing that a mixed nitrogen source enhances auxin synthesis by the shikimic acid pathway, increasing carbon and nitrogen utilization. Commonality in metabolic responses to stress is to be expected, and comparative analysis of different stresses has revealed considerable overlap (Obata et al., 2015).

Autophagy, a constitutive cellular process of homeostatic recycling, is exacerbated under nutrient deficiency. Although it is one of the most studied cell biology topics in recent times, research on autophagy in maize using metabolomics is limited. In a pioneer study that combined both, McLoughlin et al. (2018) applied a multi-omics approach to nitrogen-starved maize and identified numerous metabolic alterations, mainly in lipid and secondary metabolism.

Organochlorine pesticides were shown to alter the glycolysis/gluconeogenesis balance, inactivate the TCA cycle, redistribute nitrogen compounds, and increase fatty acid production and oxidation in maize roots (Blondel et al., 2016). Smoke, not a stressor per se but an inducer of plant persistence and recolonization after wildfires, was shown to affect carbohydrate and energy pathways in young maize roots (Çatav et al., 2018).

#### How do combined stresses affect the maize metabolome?

In the last decade, maize metabolic responses to single abiotic stresses have been relatively well documented, with studies covering salinity (Gavaghan et al., 2011; Henry et al., 2015; Richter et al., 2015; Forieri et al., 2016; Li et al., 2018), water deficit (Alvarez et al., 2008; Virlouvet et al., 2011; Witt et al., 2012; Barnaby et al., 2013; Benevenuto et al., 2017; Yang et al., 2018b), cold (Noblet et al., 2017; Li et al., 2019c; Urrutia et al., 2021), and high temperature (Suwa et al., 2010; Sun et al., 2016a). However, in natural habitats, plants often experience a combination of stress conditions, and the effects of these interactions on the maize metabolome is less well characterized. The plasticity of maize molecular responses to combinations of different abiotic stresses has received some attention due to their potentially highly damaging effect compared with isolated stress conditions. For instance, maize plants subjected to a combination of water deficiency with salt or heat stress presented metabolic responses distinct from those of plants subjected to one stress alone, suggesting that maize exhibits metabolic plasticity in response to different stress conditions (Sun et al., 2015, 2016b). The metabolome analysis of plants grown under elevated CO_2_ and subjected to sudden heat shock stress identified malate metabolism as a key player in the recovery of photosynthetic activity after a short-term heat wave (Qu et al., 2018). In addition, elevated CO_2_ was shown to eliminate early responses of maize leaf metabolites under water deficit (Sicher and Barnaby, 2012). Moreover, GC-MS-based metabolite profiles of leaves from 10 tropical maize hybrids with diverse abiotic stress tolerances were analyzed after exposure to drought, heat, and both stresses simultaneously in field trials (Obata et al., 2015). Interestingly, most metabolic changes in the combined treatment (drought and heat) could be predicted from the sum of responses to the individual stresses. This study also identified metabolite signatures closely related to grain yield under abiotic stress conditions, specifically highlighting *myo*-inositol and raffinose as promising metabolic markers for breeding purposes.

The metabolic changes in maize plants caused by combined abiotic and biotic stress treatments remain largely unknown. A few studies have recently touched upon this interesting topic, revealing that different abiotic stresses can have distinct effects on biotic threats. For instance, elevated CO_2_ was observed to reduce phytoalexin accumulation, enhancing the mycotoxigenic effects of *Fusarium verticillioides* in maize (Vaughan et al., 2014). However, when combined with drought stress, high CO_2_ levels increased phytoalexin content, thereby enhancing maize phytochemical defenses against *F. verticillioides* (Vaughan et al., 2016). Elevated CO_2_ alone was also shown to compromise maize metabolic defenses against *Aspergillus flavus* by reducing the levels of the keto-acidic sesquiterpenoid zealexin A4 (Christensen et al., 2018a). Additive and synergistic effects of flooding and anti-insect defense responses were also observed against *Spodoptera frugiperda* (fall armyworm) infestation in maize. In this case, the combined stress led to elevated production of salicylic acid, which did not occur in the individual stresses, resulting in extra salicylic acid-dependent protection against *S. frugiperda* (Block et al., 2020). Moreover, the combination of flooding and herbivory led to a remodeling of the phenylpropanoid pathway, which in turn increased maysin accumulation by 2-fold compared with the control non-infested plants (Block et al., 2020). Interestingly, heat stress applied prior to fungal inoculation had a negative effect on maize resistance to *Cochliobolus heterostrophus*, and targeted metabolome analysis revealed that deficiency in the hydroxycinnamic acid p-coumaric acid may have contributed to the observed heat-induced susceptibility to the fungus (Christensen et al., 2021). These findings highlight the phenotypic variation observed in maize plants under different stress combinations and demonstrate the complexity of plant–environment relationships. More intriguingly, they point to the fact that abiotic stresses can also predispose crops to more severe biotic threats.

### Changes in the maize metabolome under beneficial biotic interactions

Plant–microbe interactions are ubiquitous and are important for the health of plants and soil. Many of these interactions occur in the rhizosphere and often result in positive effects when plants associate with beneficial microorganisms such as plant-growth-promoting bacteria, mycorrhizal fungi, rhizobia, and endophytes (Kaur and Suseela, 2020).

Growth-promoting bacteria of the *Azospirillum* and *Bacillus* genera are the most studied in maize. Walker et al. (2011) were the first to show an effect of growth-promoting bacteria on the plant secondary metabolome. Two subsequent studies demonstrated that *Azospirillum*-mediated signals pass through the xylem (Rozier et al., 2016) and that *Azospirillum*-mediated yield enhancement may be manifested mainly in increasing the chance that seeds turn into adult plants (Rozier et al., 2017). An interesting aspect of the bacteria–maize interaction is the action of growth-promoting bacteria in the context of nutrient availability. This was investigated by Rozier et al. (2017) under field conditions and in a more recent study using a variety of soil properties or fertilization modes (Vinci et al., 2018). Vinci et al. (2018) showed that the combination of *Bacillus amyloliquefaciens* inoculation with composted organic phosphorus fertilizers not only increased plant phosphorus and nitrogen uptake but also had a greater impact than mineral fertilizers on the plant metabolome. Furthermore, the influence of growth-promoting bacteria on the metabolome of different maize strains, lines, and cultivars was also addressed in some of the aforementioned studies by comparing growth-promotion-responsive strains with non-responders (Walker et al., 2011; Rozier et al., 2016). In a later study, two genetically distant inbred lines were inoculated with two free-living atmospheric nitrogen (N_2_)-fixing bacteria (*Herbaspirillum seropedicae* and *Azospirillum brasilense*), also known as diazotrophic bacteria, or their counterparts deficient in nitrogenase activity (Brusamarello-Santos et al., 2017). This study suggested that leaf-level variations in some metabolites can occur during plant-bacterial interaction irrespective of N_2_ fixation, but changes in specific metabolites, such as mannitol, trehalose, and isocitrate, seem to be specific for the N_2_ fixation capacity of the two studied bacteria, pointing to these metabolites as putative markers for the interaction with diazotrophic bacteria. The action of growth-promoting bacteria and fungi in the context of contaminated soils also constitutes a niche subtopic of crucial ecological importance. The colonization of maize grown in nutrient-poor mining-affected soil was shown to improve nutrient uptake and alleviate heavy-metal stress (Li et al., 2014; Dhawi et al., 2015).

Although the above studies are important, the arbuscular mycorrhizal fungi–plant interaction is the most ancient and widespread plant mutualistic association, affecting ∼80% of land plants and most cultivated plants, including maize. The fungi facilitate plant uptake of mineral nutrients, mainly phosphorus and nitrogen, by increasing the absorbing surface area in exchange for a carbon source essential for fungal growth (Wang et al., 2017). Arbuscular mycorrhizal colonization of maize roots affects the metabolome in both roots and shoots, conferring beneficial effects on plants under abiotic stresses, including water deficit and salinity (Sheng et al., 2011; Hu et al., 2020). The effects of mycorrhizal Pi uptake cause several changes in leaf metabolism, including changes in carbon versus nitrogen metabolism in leaves that preferentially take up Pi via mycorrhiza and the concomitant induction of systemic defense and accumulation of secondary metabolites, suggesting that the priming effect observed in maize leaves is a mycorrhiza-specific response (Gerlach et al., 2015).

## Genetically modified maize

Approximately a third of the worldwide maize crop area is dedicated to genetically modified (GM) maize (OECD, 2018). As part of the safety assessment that GM crops undergo before introduction to the market, so-called substantial equivalence to wild-type counterparts must be shown. For decades, targeted analysis of several key metabolites has been the standard. The bias and limitations of this approach are clear, and it is gradually being replaced with non-targeted analyses. One of the first such studies in maize used the insect-resistance gene *Cry1Ab* as a model and employed for the first time the partial least square discriminant analysis (PLS-DA) statistical method (Manetti et al., 2006), which has since appeared in countless metabolomics publications. Another early example indicated that, for the transgenic events studied, (1) metabolites not included on the list of key metabolites to be tested showed much higher variability than those recommended by the OECD (Harrigan et al., 2007); and (2) the impact of GM trait insertion on the grain metabolome variation in hybrids derived from the glyphosate-tolerant maize strain NK603 was negligible compared with corresponding GM trait-negative hybrids, supporting the hypothesis that residual genetic variation due to the conventional breeding process accounted for the observed differences between GM and non-GM segregants (Harrigan et al., 2016). However, comprehensive non-targeted metabolomics revealed that the NK603 strain was not substantially equivalent to its nearest isogenic non-GM strain, DK2675 (Mesnage et al., 2016). An earlier study assessed the contributions of two genetic modifications (glyphosate tolerance and *Bacillus thuringiensis* insect resistance) relative to those of environmental factors such as growth conditions and location (Frank et al., 2012). The authors concluded that most differences were related to natural variability rather than to genetic modification, with a substantial contribution from environmental factors. A similar conclusion was reached in a study of stacked GM maize that combined insect resistance (*Cry*) with glyphosate tolerance (*Epsps*) genes in a single strain (Wang et al., 2018b). Although these studies provided important information that could inform regulatory frameworks established to determine whether transgenics could generally be regarded as safe, it must be borne in mind that such studies must be performed empirically for each specific transgenic event, and one cannot merely generalize from them.

GM is mediated, among other things, through transformation into embryonic callus, a tissue that is ideal for this purpose thanks to its inducible totipotency. However, the relatively low rate of embryonic callus induction and regeneration in maize has hampered genetic engineering in this species. A recent study offers one of the most comprehensive investigations to date of the factors that control this process in maize, combining proteomics and metabolomics of different lines and induction stages (Ge et al., 2017). These analyses revealed that differences in the capacity to produce embryonic callus involve various metabolic pathways. The induction of amino acids, lipids, and sugar metabolism, as well as the regulation of hormone synthesis, including that of auxin, cytokinin, jasmonic acid, and brassinosteroids, seems to be associated with a higher rate of embryonic callus induction.

In the last decade, the RNA-guided CRISPR/Cas9 (clustered regularly interspaced short palindromic repeats/CRISPR-associated protein 9) system has been applied to plant genome editing and represents a massive breakthrough. The CRISPR/Cas9 system introduces stable mutations at specific sites dictated by a single guide RNA, with a much cleaner genetic background. It has been increasingly used in maize studies and is a great advance for both functional research and breeding of maize (Liu et al., 2020a). Despite the great advances in gene discovery and trait development in crops brought about by CRISPR/Cas9, this technology shares major challenges with classic genetic modification methods, e.g., polyploidy, transformation efficiency, and tissue regeneration, which remain concerns in the development and application of crop genome editing (Mao et al., 2019). Due to its ability to determine global metabolic changes, metabolomics has been proposed as a route for the identification of gene-edited individuals (Fraser et al., 2020). Although this is clearly dependent on the gene-editing event having metabolic consequences, there is no doubt that metabolomics can help to discriminate gene-edited from non-edited plants during regulatory assessment to identify both intended and unanticipated detectable metabolic outcomes.

## Integration of omics data

Rapid advances in omics technology, together with falling costs, especially of sequencing, have led to the parallel application of several omics techniques in many studies. In this regard, parallel application is distinguished from *bona fide* data integration. In the former, metabolomics is accompanied by either proteomics (Ge et al., 2017), transcriptomics (Xu et al., 2019), or both (Barros et al., 2010; Casati et al., 2011; Amiour et al., 2014; McLoughlin et al., 2018). An important example is the proteome and metabolome profiles of glyphosate-tolerant GM maize, which show that despite previous claims, it is not in fact equivalent to its isogenic counterpart in protein and metabolite content (Mesnage et al., 2016). In studies that implement *bona fide* integration, data from the different sources are mathematically combined, often in the form of network analysis, to unravel correlations that are not otherwise immediately apparent (Xu et al., 2019). The use of metabolomics alongside more exotic omics datasets has also gained attention; these include fluxomics, which determines the rates of metabolic reactions (Cocuron et al., 2019); exometabolomics, also known as metabolic footprinting, which studies changes in extracellular metabolites (Zha et al., 2014); and ionomics, the analysis of mineral nutrient and trace element composition (Guo et al., 2017).

The integration of phenotypic data, mostly relevant agronomic traits, with the metabolome is present in several studies and goes hand in hand with the aim of yield improvement. For instance, in a seminal study, GC-MS-based metabolite profiling was integrated with enzymatic activity profiles of 29 key central-metabolism enzymes, as well as agronomic traits (Cañas et al., 2017). This study, conducted on a panel of 19 genetically and geographically distant maize lines, yielded a “maize ideotype,” a hypothetical strain optimized for high yield, and several metabolites were identified as excellent predictors of kernel size, e.g., chlorogenates.

The optimal methodology for integrating omics data for hybrid selection and prediction has been thoroughly tested and discussed. It has been shown that genomic, and more so transcriptomic, data alone are superior to metabolomic data (proteomics was not included) with regard to predictive power for complex and highly heterotic traits (Westhues et al., 2017). Despite this fact, Westhues et al. (2017) still acknowledge the potential (and practical) contribution of metabolites, by force of their “physiological proximity to the phenotype, which provides information that is impossible to infer from DNA or proteins.” In this context, it is important to mention a study that assayed the robustness of metabolite levels in inbred lines versus hybrids using metabolomics alone. The hybrids displayed greater robustness, underscoring the much-studied phenomenon of heterosis and providing a predictive model for the performance of new hybrids (de Abreu E Lima et al., 2017). In their study, Amiour et al. (2012) stated: “It was also found that the integration of the three 'omics' studies is not straightforward, since different levels of regulation seem to occur in a stepwise manner from gene expression to metabolite accumulation.” With this in mind, considerable achievements have been made using omics integration. A notable example is the study by Rao et al. (2014), which provided the research community with a comprehensive metabolic map of maize kernels. Non-targeted profiling conducted on 14 representative maize lines showed that several metabolites could be used to distinguish between the lines.

Some initiatives, namely OPTIMAS-DW (http://www.optimas-bioenergy.org/optimas_dw; Colmsee et al., 2012), MaizeGDB (https://maizegdb.org/; Andorf et al., 2015), MODEM (http://modem.hzau.edu.cn/; Liu et al., 2016a), and most recently ZEAMAP (http://www.zeamap.com/; Gui et al., 2020), have aimed to integrate maize omics results, including metabolomics, in more comprehensive databases, providing useful tools for the search, analysis, and visualization of these rich datasets. In addition, since the development of the C4GEM, an early attempt to build a genome-scale metabolic model to study C_4_ metabolism (Dal'Molin et al., 2010), some *in silico* reconstructions of maize metabolism have been presented as ideal tools for the integration of different omics approaches. For instance, the *Zea mays i*RB1563 model comprises 1,563 genes and 1,825 metabolites that participate in 1,985 reactions from both primary and secondary metabolism. It revealed unique reactions and metabolites compared with the AraGEM model for *Arabidopsis* and the C4GEM (Saha et al., 2011). A second-generation genome-scale metabolic model for the maize leaf, approximately four times broader than the earlier *i*RS1563, was generated to capture C_4_ carbon fixation and investigate nitrogen assimilation by modeling the interactions between the bundle sheath and mesophyll cells (Simons et al., 2014). Moreover, a tissue-specific metabolic model (Seaver et al., 2015) and one that describes mesophyll and bundle sheath cells in different segments of the developing maize leaf (Bogart and Myers, 2016) have substantially increased the accuracy of predictions of the spatial variation in metabolic state and metabolic fluxes from expression data.

## The origin, evolution, and natural variation of maize through a metabolomic lens

Similar to other crops with a long history of human cultivation, many favorable genes that were lost during the domestication of maize, including those related to nutritional value and stress tolerance, remain hidden in wild ancestors. Therefore, much research has been dedicated to understanding the origin, evolution, and natural variation of maize. Pioneering studies have reported the metabolite profiling of various maize strains, lines, and crosses derived from or grown in different locations, demonstrating the considerable impact of origin on metabolic composition (Röhlig et al., 2009; Skogerson et al., 2010).

The influence of teosinte on the genetics, ecology, and composition of domesticated maize has also attracted considerable attention. For instance, a large-scale metabolite-based quantitative trait loci (mQTL) analysis in a population generated from crossing teosinte with the maize inbred line Mo17 demonstrated massive metabolic variations (Li et al., 2019b). Most of the metabolites analyzed displayed an additive effect in the presence of alleles from the teosinte genome, whereas the opposite pattern was observed for grain yield and shape trait quantitative trait loci (QTL). Another comprehensive metabolomic analysis was conducted on teosinte and tropical and temperate maize, as well as on a teosinte ⨉ maize cross. Lipids, alkaloids, and terpenoids mostly differed between teosinte and tropical maize, whereas benzoxazinoids differed between tropical and temperate maize. Further integration with transcriptomics led to the identification of several genes responsible for the metabolic divergence (Xu et al., 2019). The genetic architecture of oil and carotenoid traits in a teosinte-maize population has also been targeted. A trait-QTL network was constructed to assess the genetic relationships among 33 oil- and carotenoid-related traits. The evolutionary trajectories of the genes or QTLs responsible for variations in oil and carotenoid traits revealed that these traits caused diverse selection events during maize domestication. This indicates the complex selection patterns of the genes that underlie maize kernel nutritional traits and shows that teosinte alleles can also be valuable for improving those traits (Fang et al., 2020).

In fact, most agriculturally and economically important traits have a complex genetic basis (i.e., they are determined by multiple QTLs); therefore, precisely locating and characterizing the functional loci are extremely important for crop improvement (Wen et al., 2016a; Liu et al., 2020b). Linkage mapping based on an F2 or recombinant inbred line (RIL) from crosses between two or more parental accessions is a well-known approach for locating QTLs. However, only a few QTLs are usually detected by linkage mapping in each experiment; further fine mapping to obtain a more precise genetic position is needed, and larger secondary populations are required to achieve sufficient map resolution (Xiao et al., 2017). Even with the introduction of high-density maps generated by next-generation sequencing, which increase the mapping resolution of mQTLs, this approach is not scalable for exploring variation in abundant diverse germplasm (Luo, 2015).

In this context, genome-wide association studies (GWASs) have been used in diverse populations as a strategy for fine mapping QTLs. Although the first association mapping study in maize was performed about 20 years ago (Thornsberry et al., 2001), only considerably later was this approach used to study maize metabolism, revealing that allelic variance in *FAD2*, which encodes a fatty acid desaturase, is responsible for differences in the oleic acid content of kernels (Beló et al., 2008). Since then, GWAS in plants has undoubtedly gained popularity, and maize is no exception (Xiao et al., 2017). The diploid genome and the cultivation history of maize account for the impressive phenotypic diversity that lends itself to association studies.

In recent years, GWAS and linkage analysis have been successfully conducted to dissect the diversity of maize metabolic traits in populations of either natural variation (accessions) or generated variation (introgression lines, RILs, and backcross inbred lines) (Table 1). Metabolite-based GWASs (mGWASs) are classic applications of omics integration in which metabolite levels, serving as quantitative traits, are correlated with genomic marker data (single nucleotide polymorphisms [SNPs]) from a population. One of the major advantages of mGWAS is its ability to help identify novel genes in metabolic pathways. Due to the large diversity across experimental populations and precise evaluation of metabolite levels, it is much easier to identify the genetic variants that control the accumulation of metabolites rather than QTLs related to agricultural performance, which usually have a moderate or low effect (Fang and Luo, 2019). However, factors such as the degree of diversity in the population, the density and quality of the SNP data, and the tissue selected for metabolite profiling may greatly influence the results.

Natural variation in the maize metabolome has been widely explored by mQTL research (Liu et al., 2020b). For instance, the Goodman Diversity Panel (Flint-Garcia et al., 2005) has been used numerous times in various studies. The panel consists of 302 accessions and captures a large proportion of the alleles in cultivated maize. GWAS analyses focusing on vitamin E in maize kernels demonstrate how consecutive studies build upon each other with incremental but significant improvements, with, in this case, the expansion of the profiled metabolites (from only several tocopherols to all tocochromanols), the use of better populations, and, in the final study, the comparison of two different populations created specifically to reflect two different vitamin E traits (Li et al., 2012; Lipka et al., 2013; Diepenbrock et al., 2017; Fenton et al., 2018). In addition, non-targeted metabolite profiling identified almost 4,000 metabolic features in leaf bases and tips (Zhou et al., 2019) and found an interesting bimodal metabolite distribution. The vast majority of metabolites were present in less than half the lines, bases and tips differed in flavonoid content, and different maize varieties differed in benzoxazinoid content. The analysis of root volatiles and the application of mGWAS enabled the identification of the *terpene synthase21* (*tps21*) gene as an important player in a previously unrecognized β-costic acid pathway in maize that contributes to fungal pathogen resistance (Ding et al., 2017). Later, an integrative study used association mapping, pan-genome multi-omic correlations, enzyme structure-function, and targeted CRISPR-Cas9 mutations to identify genes involved in hormone pathways that partition diterpenoid defenses (Ding et al., 2019). Kauralexin biosynthesis was shown to use *ent*-isokaurene formed by diterpene synthases recruited from gibberellin metabolism. This mechanism minimizes the unregulated accumulation of gibberellin precursors, which could affect hormone signaling during the defense response to biotic stress (Ding et al., 2019). Using the same approaches, Ding et al. (2020) also identified 10 genes in three zealexin gene clusters that encode four sesquiterpene synthases and six cytochrome P450 proteins. The findings from this elegant work suggest a so-called hourglass-shaped biosynthetic network in maize defensive terpenoid metabolism in which terpene synthase-derived metabolites meet at a single cytochrome P450 monooxygenase enzyme node, with subsequent diversification via pathway-specific enzymes (Ding et al., 2020).

Another important population used for the genetic mapping of metabolic traits in maize is the MaizeGo panel (http://www.maizego.org/Resources.html), consisting of 540 lines (Yang et al., 2011). mGWAS has been applied in studies using this population or a subset of this population, and several QTLs and, subsequently, genes have been identified. For instance, the identification of two insertion/deletions within a gene encoding γ-tocopherol methyltransferase (*ZmVTE4*) and an SNP located ∼85 kb upstream of this gene revealed that *ZmVTE4* is a major gene involved in natural phenotypic variation in α-tocopherol of maize kernels (Li et al., 2012). Later, a combination of linkage and association analyses suggested a role for non-tocopherol pathway genes in the modulation of natural tocopherol variation, including genes involved in fatty acid metabolism, chlorophyll metabolism, and chloroplast function (Wang et al., 2018a). The genetic basis for natural variation in oil biosynthesis, fatty acid composition (Li et al., 2013), and amino acid metabolism in kernels has also been examined in this population (Deng et al., 2017). Broader analyses of the maize metabolome in different tissues have identified novel genes involved in key processes in the formation of phenolamides and flavonoids (Wen et al., 2014), as well as variations underlying the trehalose, aspartate, and aromatic amino acid pathways (Wen et al., 2018).

Recently, some maize studies have used a new approach that combines metabolic pathway analysis with GWAS to determine the cumulative effects of several genes clustered according to their shared biological function. This approach can potentially find novel clues to the genetic basis of a trait by revealing biological insights that may not appear when focusing on only one or a few genes that are most significantly associated with a certain trait (Li et al., 2019a). The pathway-based approach, first developed to study human disease, has just begun to be applied to plants and maize. It has been used specifically to study corn earworm resistance (Warburton et al., 2018) and lipid biosynthesis (Li et al., 2019a). It seems likely, based on the success of these studies, that this approach will gain further utility in maize and indeed in other species.

## Concluding remarks

Recent years have been characterized by impressive advances in the identification of maize metabolites. Despite this progress, as in general for the plant metabolome, it is clear that the majority of maize metabolites cannot be accessed by current profiling methods. Therefore, metabolite identification remains one of the main challenges in metabolomics, regardless of the organism analyzed. As described in this review, several metabolomics studies have performed targeted analysis of specific metabolites or classes, in which case standards can often be used to confirm metabolite identities. Other cases of a more comparative nature focus on features or peaks (their existence, absence, or patterns of abundance across different plant lines), obviating the need for metabolite identification (Baniasadi et al., 2014). Moreover, the integration of publicly available metabolomics data (see section “integration of omics data”) is currently proving highly informative, with network analysis such as that carried out using Global Natural Product Social Molecular Networking (GPNS) being applied to LC-MS (Wang et al., 2016) and more recently GC-MS (Aksenov et al., 2020) datasets.

In addition to the identification of new metabolites, the spatial distribution of already known metabolites within organisms has also been of special interest. For instance, as a C_4_ plant, maize spatially separates its photosynthesis in a well-characterized mechanism for concentrating CO_2_ in the bundle sheath cells (Figure 1). The operation of such a two-cell pathway and the dynamic subcellular compartmentalization of the maize metabolome underscore the usefulness and need for the development of imaging methods such as MALDI-MSI (see section “methodologies”) that will help us better understand metabolic compartmentalization in this species. The further development of single-cell metabolomics is likely to advance our understanding of how cells work in concert to achieve organismal function.

Metabolomics approaches have been used as an important tool for the identification and functional characterization of metabolism-associated genes in maize (Table 1). The combination of metabolomics and quantitative genetics approaches represents a highly powerful instrument for characterizing the genetic architecture associated with the accumulation of metabolites that are important for plant performance or for the biofortification of maize for human and animal use. The analysis of mutants for candidate genes, linkage mapping, and mGWAS have been used extensively for this purpose. Of particular note in this light is that improving the nutritional composition of what is now the major crop worldwide will be greatly facilitated by metabolomics studies of maize kernels from broad populations and biofluid metabolomics of human cohorts. It will ultimately be possible to better distinguish nutritional benefits by comparing individuals who eat maize of a particular type with those who do not and determining whether this corresponds to their relative incidence of chronic disease.

Continued advances in functional genomics and genetics, the design of new, highly genetically diverse maize populations, and the characterization of the pan-panicoid metabolome should be targets in the near future. This will certainly deepen our understanding of maize metabolism and evolution, subsequently contributing to the improvement of maize toward a more ideal crop by either the *de novo* domestication of wild relatives, as recently proposed by Fernie and Yan (2019), or by the manipulation of only a specific set of genes.

## Funding

This work was supported by the 10.13039/501100002347Bundesministeriums für Bildung und Forschung (BMBF, German Federal Ministry of Education and Research) under the FullThrottle (031B0205B) and Reconstruct (031B0200E) projects.
